# Distributional Response of the Rare and Endangered Tree Species *Abies chensiensis* to Climate Change in East Asia

**DOI:** 10.3390/biology11111659

**Published:** 2022-11-13

**Authors:** Peng-Bin Dong, Li-Yang Wang, Ling-Juan Wang, Yun Jia, Zhong-Hu Li, Gang Bai, Rui-Ming Zhao, Wei Liang, Hong-Yan Wang, Feng-Xia Guo, Yuan Chen

**Affiliations:** 1 College of Agronomy, College of Life Science and Technology, State Key Laboratory of Arid Land Crop Science, Gansu Agricultural University, Lanzhou 730070, China; 2 College of Agriculture, Ningxia University, Yinchuan 750021, China; 3 Xi’an Botanical Garden of Shaanxi Province (Institute of Botany of Shaanxi Province), Xi’an 710061, China; 4 Key Laboratory of Resource Biology and Biotechnology in Western China (Ministry of Education), College of Life Sciences, Northwest University, Xi’an 710069, China

**Keywords:** *Abies chensiensis*, alpine species, global climate change, migration prediction, potential distribution

## Abstract

**Simple Summary:**

The adaptation, migration, and extinction of species are closely associated with climate changes and shape the distribution of biodiversity. Plants in alpine ecosystems are particularly sensitive to climate change. In recent decades, the loss and fragmentation of suitable habitats for species due to climate change have caused alpine plants to become extinct or to be replaced by other species. Thus, to predict how climate change will influence the survival and suitable habitats of the rare and endangered tree species *Abies chensiensis* in East Asia, we used a maximum entropy model to simulate the changes in its distribution area from historical periods to future periods. Our results illustrate that temperature is an indispensable factor affecting the presence and suitable habitats of *A*. *chensiensis*. In the future (the 2050s and 2070s), the suitable distribution area will contract significantly, and the migration routes of the centroids will tend to migrate toward the southern high-altitude mountains. These results may contribute to a more comprehensive understanding of potential geographical distribution patterns and the distribution of suitable habitats for some rare and endangered plant species in East Asia and may help implement long-term conservation and the reintroduction of these species.

**Abstract:**

Globally, increasing temperatures due to climate change have severely affected natural ecosystems in several regions of the world; however, the impact on the alpine plant may be particularly profound, further raising the risk of extinction for rare and endangered alpine plants. To identify how alpine species have responded to past climate change and to predict the potential geographic distribution of species under future climate change, we investigated the distribution records of *A*. *chensiensis*, an endangered alpine plant in the Qinling Mountains listed in the Red List. In this study, the optimized MaxEnt model was used to analyse the key environmental variables related to the distribution of *A*. *chensiensis* based on 93 wild distribution records and six environmental variables. The potential distribution areas of *A*. *chensiensis* in the last interglacial (LIG), the last glacial maximum (LGM), the current period, and the 2050s and 2070s were simulated. Our results showed that temperature is critical to the distribution of *A*. *chensiensis*, with the mean temperature of the coldest quarter being the most important climatic factor affecting the distribution of this species. In addition, ecological niche modeling analysis showed that the *A*. *chensiensis* distribution area in the last interglacial experiencing population expansion and, during the last glacial maximum occurring, a population contraction. Under the emission scenarios in the 2050s and 2070s, the suitable distribution area would contract significantly, and the migration routes of the centroids tended to migrate toward the southern high-altitude mountains, suggesting a strong response from the *A*. *chensiensis* distribution to climate change. Collectively, the results of this study provide a comprehensive and multidimensional perspective on the geographic distribution pattern and history of population dynamics for the endemic, rare, and endangered species, *A*. *chensiensis*, and it underscores the significant impact of geological and climatic changes on the geographic pattern of alpine species populations.

## 1. Introduction

Over the past 100 years, the average temperature in the alpine region has risen by nearly 2 °C, which is almost twice as large as the average global increase; however, the last 30 years have seen a particularly rapid rise, with frequent severe weather events [1,2]. As temperatures warm, future climate change will not only alter surface temperature and precipitation patterns [3], but will also affect ecosystems and biomes, leading to changes in the physiological metabolism, geographical distribution, and population size of plants, particularly those of alpine origin [4,5]. Previous research has shown that the rate and magnitude of warming are much greater at higher altitudes than at lower altitudes [6] and, by 2080, more than 50% of Europe’s plant species will be threatened, while more than 60% of alpine species will face extinction [7]. Based on climate data for the Tibetan Plateau recorded in recent decades and combined with Global Circulation Models (GCMs), Shi et al. suggested that the subalpine treeline on the Tibetan Plateau will rise to higher elevations under future climate change scenarios [8]. As many species migrate to higher altitudes, those with restricted geographic extents, small population sizes, or high habitat specificity (i.e., rare and endangered species) will eventually become extinct as their ranges shrink [9,10,11]. In addition, in the process of migration, alpine plant species will inevitably face fierce competition from native plants, either through their decline or through the degradation and disappearance of native plant species, resulting in a significant reduction in alpine plant diversity [12]. Therefore, paying attention to the response of rare and endangered species to climate change in the past and future is helpful not only in understanding the historical causes of species formation and changes in the geographical distribution, but is also critical for assessing biodiversity vulnerability and guiding conservation efforts.

Given global climate change, species distribution models (SDMs) have been widely used to assess the risk of invasive alien species, predict the potential distribution of species, the impact of climate change on species, and conservation strategies for endangered species [13,14]. SDMs are an effective method of assessing the ecological requirements or predicting the potential geographic distribution of rare species based on known species distribution data and relevant environmental factors, and they are widely used in ecology and biogeography [15,16]. Currently, among the many species distribution models (Domain, GARP, CART, CLIMEX, Bioclim, ENFA, and MaxEnt), MaxEnt has the superior performance [17]. The modeling has low sample size requirements, high accuracy, and stability even with small sample sizes, and is the preferred model for predicting the potential distribution areas of endangered plants [18,19,20]. Hernandez et al. (2006) constructed SDMs for 18 species in California with four different methods (GARP, Domain, Bioclim, and MaxEnt), and the results showed that the model constructed with MaxEnt performed best [18]. Elith et al. (2010) modeled the spatial distribution of 226 species in six different regions of the world and evaluated the prediction results for large and small sample sizes, concluding that the MaxEnt model had the best stability with little difference in prediction accuracy across sample sizes [19]. The model has been widely used in research areas such as the potential range prediction of species, the response of species distribution to climate change, ice age refuge projections, and invasive species control.

*Abies chensiensis* Tieghem is a relict gymnosperm endemic to China, belonging to the genus *Abies* (Pinaceae), which has a high economic and medicinal value and plays an important role in ecological balance and water conservation [21,22]. The seed cone of *A*. *chensiensis* (Firs Fruit, Pusongshi in Chinese) is one of the most commonly used crude medicines in China; it stops bleeding, regulates menstruation, is anti-inflammatory, and calms the liver [23]. *A*. *chensiensis* is very sensitive to changes in temperature and, in recent decades, its wild resources have been drastically reduced and its ecological habitat has further deteriorated due to global warming, insect pests, and the long-term effects of human activities [24,25]. Therefore, *A*. *chensiensis*, an endangered plant mainly distributed in the Qinling–Daba mountain region of China, was listed as one of the national protected plants in the second category in China [21,26]. At present, the research on the conservation of *A*. *chensiensis* has focused on seed germination and fruiting characteristics [27], geographical distribution and population dynamics [24,28], species genetic diversity [29,30], pharmacological activity, and chemical composition of the species [22,31]. However, less research has been reported on modeling the ecological niche of the species. Global climate change will lead to habitat loss and the spatial isolation of rare and endangered plants, increasing the risk of extinction [32,33]; SDMs are an appropriate tool that plays an important role in habitat, population structure, and distribution areas conservation of endangered species. Thus, understanding the impact of global climate change on *A*. *chensiensis* range shifts since the Quaternary. Knowing the response of the species to climatic change, finding the historical causes of its habitat reduction and fragmentation, and determining the impact of global warming on its geographical distribution and survival prospects will provide a scientific basis for the conservation and management of this species.

In this study, the MaxEnt model was used to predict potentially suitable areas for *A*. *chensiensis* during the last interglacial (LIG) and last glacial maximum (LGM), as well as the current period and future time horizons (the 2050s and 2070s). The paleo distribution reconstruction and climate assessment reveal the change history of the *A*. *chensiensis* distribution area, the migration routes of the centroids, and the influence of climate factors on the distribution area of this species in various periods. Ultimately, they provide targeted conservation strategies for this rare and endangered species. The specific objectives are as follows: (1) to analyse the effect of climate change since the last interglacial (including ongoing global warming) and how this has affected the population numbers and distribution range of this species; (2) to identify the main environmental variables affecting the potential suitable distribution of *A*. *chensiensis*; (3) to predict the impact of future climate change on the potential habitat of *A*. *chensiensis*.

## 2. Materials and Methods

### 2.1. Species Distribution Data

**Figure 1:**
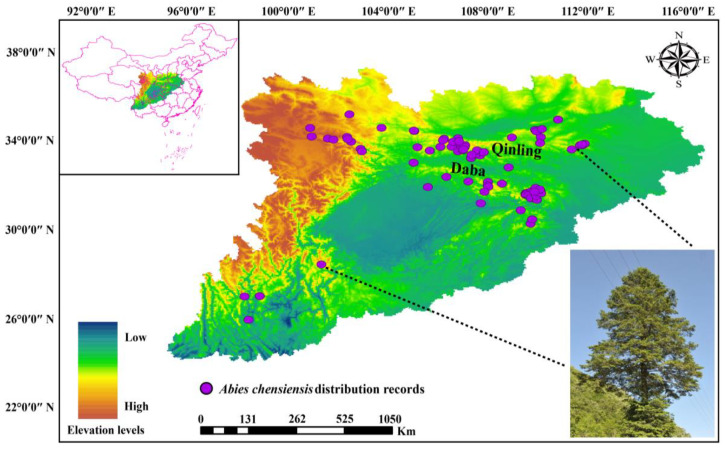
Geographical distribution of *A*. *chensiensis*. Note: purple points represent species distribution records.

Based on field surveys in the Taibai Mountains and adjacent areas, herbarium access (Chinese Virtual Herbarium, Global Biodiversity Information Facility database), and records from the literature, we obtained a total of 156 original data on the distribution of *A*. *chensiensis*. To ensure data accuracy, we removed dubious identification, cultivation, and species distribution records for which no specific geographic information was recorded, and the latitude information of each valid sampling site was recorded in detail. Spatially overlapping datapoints within the 5 km range were discarded through Google Maps, thus avoiding overfitting in the model caused by repeatedly distributed points. Finally, a total of 93 valid data distribution sites were obtained: 10 from Global Biodiversity Information Facility database, 48 from the Chinese Virtual Herbarium, 20 from the literature records, and 15 from field surveys (Table S1). ArcGIS 10.6 (https://www.esri.com/arcgis/about-arcgis (accessed on 15 July 2022)) was used to draw the geographic distribution map of *A*. *chensiensis* (Figure 1).

### 2.2. Bioclimatic Data Acquisition and Screen

Bioclimatic data were downloaded from the WorldClim database (https://www.worldclim.com/ (accessed on 22 July 2022)) and data with spatial resolutions of 2.5 arc-min were selected considering the scale of the study area. The data are the LIG (120,000–140,000 years), LGM (22,000 years), current (1970–2000) period, and future periods (the 2050s, 2070s). For the LGM period, we used paleoclimatic layers simulated by Model for Interdisciplinary Research on Climate Earth System Model (MIROC-ESM) [34]; and the Community Climate System Model Version Version 4 (CCSM4) [35]. To simulate the future distribution of *A*. *chensiensis*, BCC-CSM1.1 climate change modeling data under Representative Concentration Pathways (RCPs) 2.6, 4.5, 6.0, and 8.5, proposed by the Intergovernmental Panel on Climate Change, were used for the years 2050 and 2070 [36]. BCC-CSM1.1 has been demonstrated to be more suitable for China’s climate change characteristics, and it is recommended for use in climate change research and short-term climate prediction in China [37].

**Figure 2:**
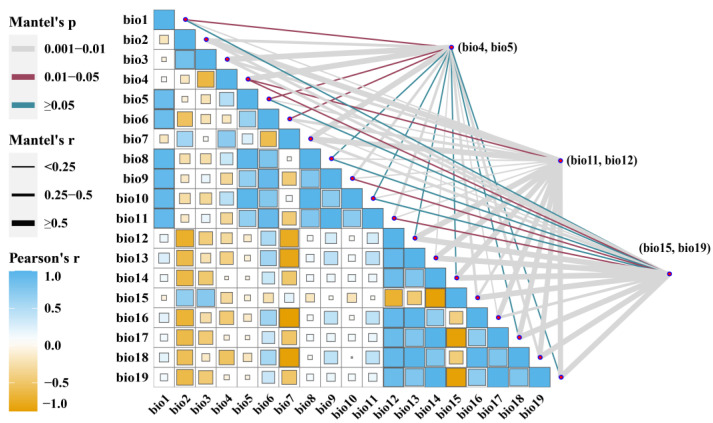
Correlation analysis of environmental variables. Blue represents positive correlations and yellow represents negative correlations.

Since there is a certain correlation between bioclimatic variables, the high correlation of variables will lead to overfitting in the model and affect its predictive ability. To avoid overfitting due to the high autocorrelation of environmental variables, the SDM toolbox was used to calculate the correlation between the 19 environmental variables (Figure 2). Environmental variables with correlation values less than 0.8 were retained, and the contribution rate of each variable was evaluated using the Jackknife method of the MaxEnt model. For environmental variables, six climate variables (Bio4, Bio5, Bio11, Bio12, Bio15, and Bio19) were selected to build the model (Table 1).

### 2.3. MaxEnt Model Operation and Evaluation

The distribution records of the *A*. *chensiensis* species and bioclimatic data were imported into the MaxEnt 3.4.1 (Princeton, NJ, USA) program for modeling analysis [38]. We started with a model built using the current distribution and current environmental variables [39]; then, past and future distributions were modeled by entering LIG or LGM and 2050 or 2070 environmental data into the projection layer window, respectively [40].

To improve the objectivity of the predictions, we randomly selected 75% of the distribution points as the training data and the remaining 25% as the test data, with the model being trained for 100 repetitions. The output data format was set to Logistic and the other values were set to default [41]. The accuracy of the MaxEnt model was evaluated by the area under the curve (AUC) value of the Receiver Operator Characteristic (ROC). AUC > 0.7 is generally considered to be a good model performance [42]. In addition, the Jackknife method was used to evaluate the weights of each variable within the model and calculated the regularised training gain, contribution, and importance of each variable to determine the major environmental factors influencing species distribution.

### 2.4. Classification of Habitat Suitability

The prediction results were imported into ArcGIS 10.6 (Californian, USA). We used the Reclassify tool in Spatial Analysis Tools to classify and visualize the suitable area and draw the potential distribution map of *A*. *chensiensis*. According to the assessment of the existence probability in the IPCC (2007), we converted the continuous suitability score (0–1) from the MaxEnt model output into a habitat distribution visualization in ArcGIS 10.6. The natural segment method was used to classify suitable areas into the following three categories: non-suitable (*p* < 0.049), low suitable (0.049 ≤ *p* < 0.196), moderately suitable (0.196 ≤ *p* < 0.441), and high suitable (0.441 ≤ *p* < 0.833), which are indicated by different colours.

### 2.5. Analysis of Centroid Migration in Suitable Distribution Areas

The SDM tool is a popular toolbox for calculating suitable species area centroid points and assessing species migration distances in latitude and longitude coordinates [43]. In this study, the SDM_Toolbox_v2.4 package (Carbondale, IL, USA) was used to calculate the position of the centroid of the species’ suitable area, and changes in the centroid position under different climate change scenarios in the LIG, LGM, current period, and 2050s and 2070s were compared. The distance of centroid migration was calculated [44]. The specific method is as follows: First, the prediction results of the potentially suitable areas of the species obtained by the simulation were converted into vector binary, i.e., the species suitability probability, *p* ≥ 0.5, was set as the total suitable area, and *p* < 0.5 was set as the non-suitable area. Then, using the Zonal Geometry statistical tool (Carbondale, IL, USA) in Spatial Analysis Tools, we selected centroid as the geometry type and obtained the position coordinates of the centroid of the suitable area of *A*. *chensiensis* under different climate scenarios in different periods.

## 3. Results

### 3.1. Accuracy of Model Analysis

The accuracy of the MaxEnt model was verified using the Receiver Operator Characteristic (ROC), which was expressed as the area under the curve (AUC). The value ranges from 0 to 1. In this study, the AUC value was selected as the index to evaluate the accuracy of the model (Figure S1). The greater the AUC value in the prediction results, the better the prediction effect, where an AUC value of 0.1–0.6 represents the failure of the prediction, 0.6–0.7 represents a poor prediction effect, 0.7–0.8 represents a moderate prediction effect, 0.8–0.9 represents a good prediction effect, and 0.9–1 represents an excellent prediction effect [45,46]. The simulation results showed that the AUC value of the test set was 0.983 in the current period (Figure S1). In our study, the AUC values of the model reached the excellent level (0.9 ≤ AUC < 1), indicating a high level of confidence in the simulation results.

### 3.2. Dominant Environmental Factors

Table S2 shows the contribution rate and importance of environmental variables to the distribution of *A*. *chensiensis* at present. In the current period, the four variables with the largest contribution rate were Bio11, Bio12, Bio5, and Bio15, with the contribution rates reaching 40.5%, 21.1%, 13.6%, and 11.8%, respectively; the permutation importance rates reached 58.5%, 15.9%, 6.9%, and 11.6%, respectively.

**Figure 3:**
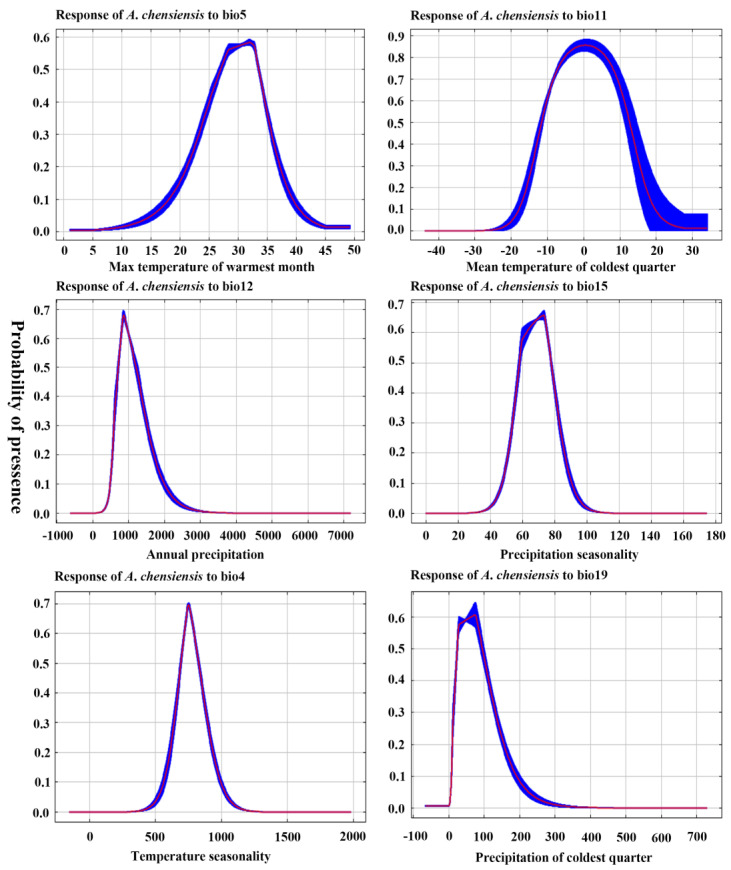
Response curves of the current existence probability of *A*. *chensiensis* to the bioclimatic variables.

The single-factor response curves of climatic factors used for the simulation were all unimodal, and the range of climatic factors with a presence probability greater than 0.5 is basically consistent with the range of climatic parameters for the highly suitable habitat. According to the response curves of the main climatic factors for *A*. *chensiensis*, the results showed that the optimum range of Bio11 was −10 to 10 °C, Bio5 was 28 to 33 °C, Bio12 was 800 to 1300 mm, and Bio15 was 60 to 78 mm (Figure 3). The Jackknife test simulations for the current period showed that the variable with the highest regularised training gain, when simulated with only one variable, was Bio11, indicating that this variable had more effective information for the simulation of the model (Figure S2). When the Bio11 variable was removed from the simulations, the variable with the greatest reduction in regularised training gain was also Bio11, indicating that this variable had information that the other variables did not, i.e., in the current period, Bio11 has a significant effect on the distribution of *A*. *chensiensis*.

### 3.3. Ecological Niche Modeling

#### 3.3.1. Suitable Areas in the Past

**Figure 4:**
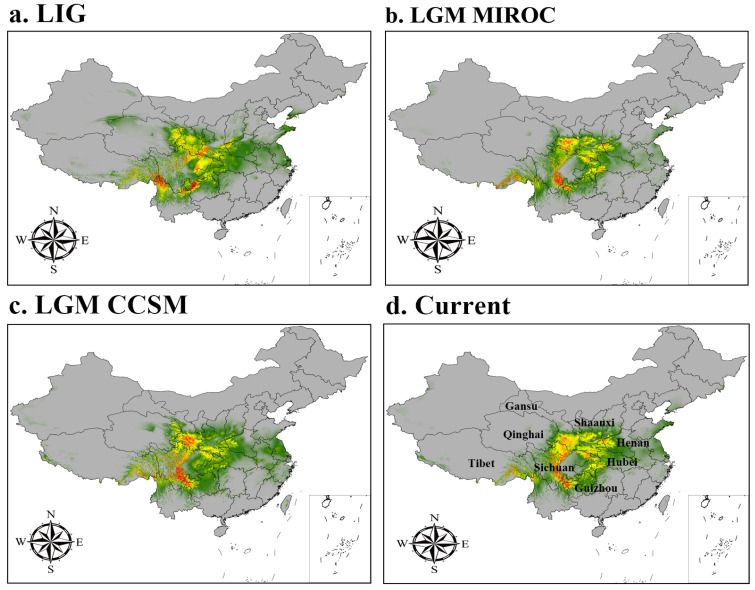
Potential distribution for *A. chensiensis* during different periods predicted by the MaxEnt model based on optimized parameters. The potential distribution of *A. chensiensis* was divided into four grades by the natural breaks method. Grey, green, yellow, and red areas represent not suitable, marginally suitable, moderately suitable, and highly suitable areas, respectively. Since the large map does not present a complete map of China, a small map is used to supplement the complete U-shaped line.

We predicted the current, LGM, and LIG distributions for *A*. *chensiensis* as shown in Figure 4. Using the natural breaks method, the potential distribution of *A*. *chensiensis* was divided into four grades: not suitable, marginally suitable, moderately suitable, and highly suitable areas. The simulation results showed that *A*. *chensiensis* had a discontinuous distribution during the LIG, with a growth range of 25° N to 37° N. Highly suitable areas were found in northern Yunnan and southern Shaanxi, moderately suitable areas were found in Ningxia and southern Gansu, and marginally suitable areas were found in Hubei. The area of the highly suitable areas decreased by 6.64 × 10^4^ km^2^, the area of the moderately suitable areas increased by 16.64 × 10^4^ km^2^, and the area of the marginally suitable areas increased by 13.73 × 10^4^ km^2^ (Table 2). In the CCSM model, the highly suitable areas were distributed in northern Gansu and southern Guizhou, the moderately suitable areas were distributed in the border area of Hubei and Shaanxi, and the marginally suitable areas were distributed in Hebei and Guizhou around the Qinling Mountains and the Huaihe River region. The distribution of the MIROC model was slightly different from that of the CCSM model. Although both models are from the LGM period, MIROC was more consistent with the expected distribution simulation for the Asian region. The current total distribution area increased by 27.79 × 10^4^ km^2^ compared with the LGM (CCSM). The size of the high suitability area decreased by 4.80 × 10^4^ km^2^, the size of the medium suitability area increased by 26.34 × 10^4^ km^2^, and the size of the marginal suitability area increased by 6.22 × 10^4^ km^2^ (Table 2).

#### 3.3.2. Current Potential Distribution Estimates

The results of the ecological niche models showed that the current period of *A*. *chensiensis* was well captured (Figure 4d). The area of the high subtility area was 35.38 × 10^4^ km^2^, the area of the medium subtility area was 48.48 × 10^4^ km^2^, and the total suitable habitat area was approximately 179.91 × 10^4^ km^2^, accounting for 18.74% of China’s territory (Table 2). Although there are some predicted areas where this species is not found, the predicted core distribution area is consistent with the current distribution records, with the higher suitability areas mainly in southern Gansu, southern Shaanxi, western Hubei, western Henan, northern Chongqing, the Sichuan basin, and nearby areas in China.

### 3.4. Suitable Distribution under Future Climate Scenarios

**Figure 5:**
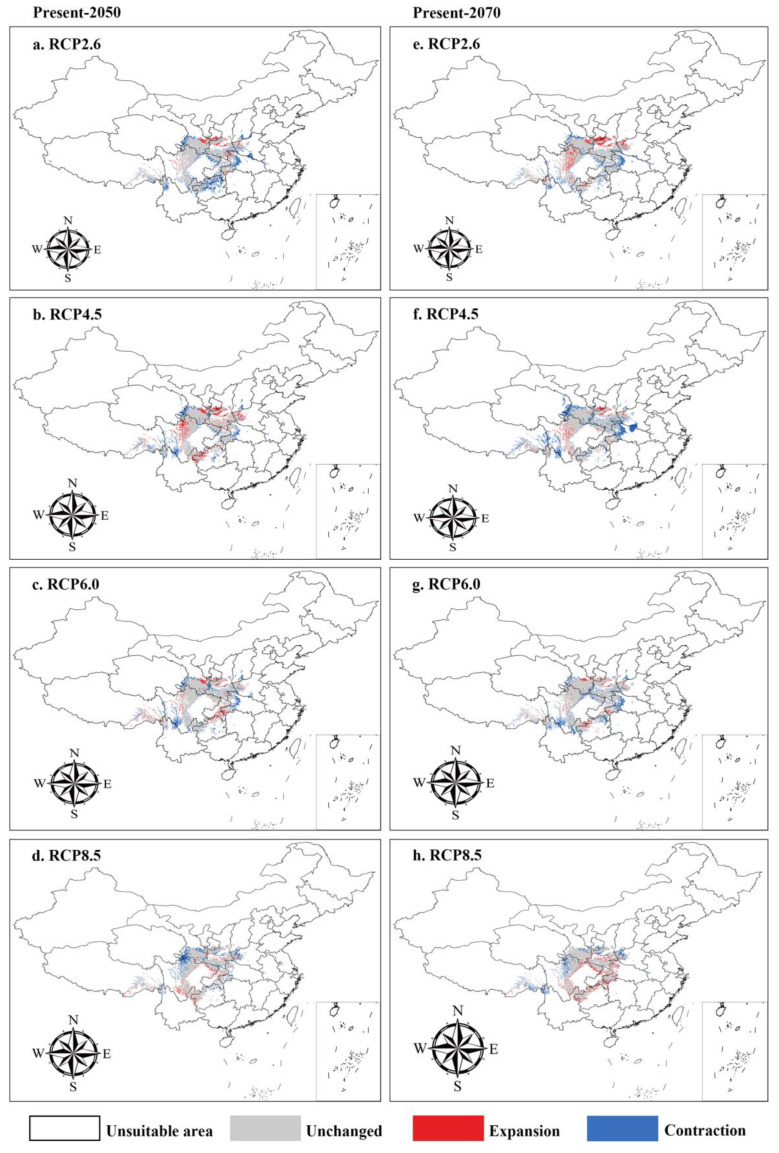
Spatial changes of *A. chensiensis* in China under emission scenarios of the 2050s and 2070s. White, Grey, Red and Blue areas represent not suitable, unchanged suitable, expansion suitable, and contraction suitable areas, respectively. Since the large map does not present a complete map of China, a small map is used to supplement the complete U-shaped line. (**a**–**d**), the 2050s; (**e**–**h**), the 2070s; (**a**,**e**), future climate scenario RCP 2.6; (**b**,**f**), future climate scenario RCP 4.5; (**c**,**g**), future climate scenario RCP 6.0; (**d**,**h**), future climate scenario RCP 8.5.

Based on the MaxEnt model, the future geographic distribution pattern of *A*. *chensiensis* was predicted. The potential habitat of the species under future (the 2050s and 2070s) climate change scenarios and the area of each habitat class were obtained. Figure 5 and Table 3 show changes in the potentially suitable areas for *A*. *chensiensis* species under four RCP climatic scenarios (RCP 2.6, RCP 4.5, RCP 6.0 and RCP 8.5). Unfortunately, under future climatic conditions, some suitable areas for *A*. *chensiensis* within China will expand, but the total distribution area will contract in the future (the 2050s and 2070s) (Figure 5 and Table 3). Compared with the current distribution, the distribution range of *A*. *chensiensis* in the 2050s will decrease in northern Guizhou, western Hubei, southern Gansu, southwestern Shanxi, northern Yunnan, and the margins of the Sichuan basin and nearby areas, and it will increase to varying degrees in southern Gansu, eastern Tibet, northwestern Sichuan, southern Shaanxi, eastern Henan, and southern Chongqing (Figure 5a–d). By the 2070s, the migration and expansion of the distribution area of *A*. *chensiensis* will basically be consistent with the results of the distribution area in the 2050s (Figure 5e–h).

### 3.5. The Migration Trends of the Geometric Center of Suitable Habitat

**Figure 6:**
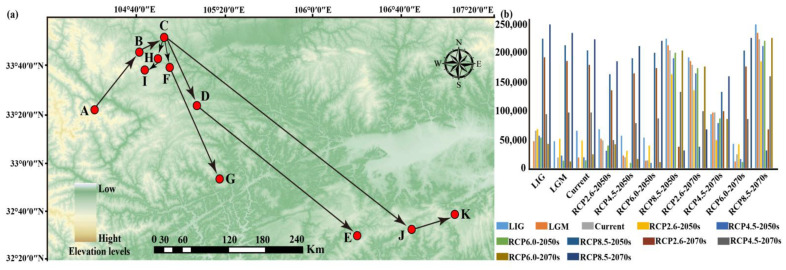
Migration of the center of suitable habitat for *A*. *chensiensis* migratory routes in historical and future climate scenarios. (**a**) Arrows indicate migratory routes and direction of the suitable habitat distribution center under historical and future climate scenarios. (**b**) The bar chart represents the core distribution shift distance for *A*. *chensiensis* under different scenarios/years. Among them, the meaning of the letters are as follows: (A) LIG; (B) LGM; (C) current; (D) RCP2.6-2050s; (E) RCP2.6-2070s; (F) RCP4.5-2050s; (G) RCP4.5-2070s; (H) RCP6.0-2050s; (I) RCP6.0-2070s; (J) RCP8.5-2050s; (K) RCP8.5-2070s.

In this study, the SDM_Toolbox in the ArcGIS 10.6 software was used to calculate the centroid distribution of *A*. *chensiensis* suitable distribution areas under different climatic scenarios and periods to obtain the centroid migration trajectory (Figure 6). At present, the centroid of the suitable distribution area is located in Luoyu Town, Xihe County, Longnan City, Gansu Province (105.288054 E, 33.772169 N). The distribution centers of *A*. *chensiensis* during the LIG and LGM were located in Lianshui Town, Gansu Province (104.774551 E, 33.347625 N) and Gansu Taishihe Town (105.101618 E, 33.689265 N), respectively (Figure 6a). In addition, compared with to the present distribution, the center of the suitable distribution area of this species in China will move to the southwest and southeast in the future (the 2050s and 2070s) (Figure 6a). By the 2050 RCP2.6, RCP4.5, RCP6.0, and RCP8.5 scenarios, the distances between the predicted distribution center and the current suitable distribution center will be 49,508 m, 19,785 m, 14,276 m, and 210,482 m, respectively; by the 2070 climate change scenarios (i.e., RCP2.6, RCP4.5, RCP6.0, and RCP8.5), the distances between the predicted distribution center and the current suitable distribution area center are 185,120 m, 99,771 m and 24,772 m, and 230,676 m, respectively (Figure 6b).

## 4. Discussion

### 4.1. Response of Species Distribution to Climate Change

Climate changes exert a significant influence on the geographical distribution of organisms. Consequently, the distribution patterns of species can reflect climatic conditions [47,48,49]. Pandey et al. conclude that the species distribution pattern of gymnosperms in China was mainly influenced by energy–water dynamics [50]. In this study, the Jackknife test of the Maxent model was used to analyse the bioclimatic variables that affect the distribution pattern of *A*. *chensiensis*. The results suggest that the mean temperature of the coldest quarter, the maximum temperature of the warmest month, the annual precipitation, and the precipitation seasonality (CV) are the main factors affecting the potential geographical distribution of *A*. *chensiensis*, with mean temperature in the coldest season making the greatest contribution rates and permutation importance. Among various climatic variables, the mean temperature of the coldest quarter and maximum temperature of the warmest month were the dominant factors influencing the spatial distribution of species during the LIG period. For the LGM period, the most dominant climatic factors in distribution were the mean temperature of the coldest quarter and annual precipitation. During this period, the climate became dry and cold, with temperatures 6 °C lower than at present and intense winter cooling [51]. The MIROC model suggested the habitats available during the LGM were relatively limited compared with the CCSM model. However, both showed that core distribution areas are located in southern Gansu and the Southwest Sichuan Basin. The distribution area of *A*. *chensiensis* increased during the LGM (CCSM) period. This expansion phenomenon during the LGM can also be found in other psychrophilic (cold-loving) plant taxa, such as *Picea likiangensis* [52], *Taxus wallichiana* [53], and *Tsuga dumosa* [54]. In our study, the prediction results show that suitable areas for the species were greatest during the LGM (Table 2). It is clear that the low temperatures of the ice age did not cause a reduction in the geographical distribution ranges of all plants but provided the conditions for the expansion of some hardy plants.

The results of the current climate analysis show that the determinantal factors affecting distribution were, in sequence, the mean temperature of the coldest quarter, annual precipitation, and the maximum temperature of the warmest month. Since the 1880s, the average annual air temperature has increased by 1.8 °C as a result of increased greenhouse gas emissions [55], with a significant impact on the mean temperature of the coldest quarter and the maximum temperature of the warmest month [36]. In some areas of the warm temperate subtropical transition zone of China, the coldest quarter suitable growth temperature (−10 to 10 °C) for *A*. *chensiensis* is exceeded, annual precipitation is less than 800 mm, and the maximum temperature of the warmest month is higher than 28 to 33 °C (Figure 3), which may be the climatic cause of the reduction in the distribution area of this species and the impact on suitable habitat. The results of this study are consistent with previous studies showing that *A*. *chensiensis* is more adapted to humid, warm climates and poorly tolerant of cold and drought characteristics [56].

The transition zone between the mid-subtropical and warm temperate zones of China has a mild climate with abundant sunshine and rainfall [57]. *A*. *chensiensis* has to receive at least 800 mm of annual precipitation and a mean temperature during the coldest quarter (−10 to 10 °C), which makes it difficult for the species to expand north of the Qinling Mountains due to temperature and precipitation limitations. This is one of the reasons why the species currently has only a sporadic “island” distribution in the Qinling and Bashan Mountains and their adjacent areas, as well as in the high-altitude areas of Shennongjia. The vegetation of the Qinba mountains is dominated by evergreen, deciduous, broad-leaved mixed forests; as an ancient relict gymnosperm, *A*. *chensiensis* has difficulty competing with dominant species and, through unique environmental adaptation strategies, is distributed in the 2300 to 3000 m subalpine zone [57]. Our study shows that the species grows in the high mountains with humid and shady areas and that it grows well in the *Indigofera amblyantha–Carex tristachya* association, which is consistent with the model’s predictions [28,58].

### 4.2. Climate Change Impacts on the Spatial Distribution Patterns of A. chensiensis

Climate change is the main driver of changes in plant distribution [59], and the recurrence of glacial and interglacial periods is considered to be the main event affecting the distribution of current plants [47]. The species distribution model (SDM) combines geographical distribution data with paleoclimate models to predict the geographical range of species distribution, which provides the possibility of exploring the impact of paleoclimate events on changes in plant distribution areas [60]. The results of the ecological niche models showed that the current period of *A*. *chensiensis* was well simulated (Figure 4d). Although there are some predicted areas where this species was not found, the predicted core distribution area is consistent with the current distribution records, with the higher suitability areas mainly in southern Gansu, southern Shaanxi, western Hubei, northern Chongqing, Sichuan basin, and nearby areas in China. During the LGM, the global temperatures were 5–12 °C lower than today, and the glacier areas were 8.4 times greater than those of the current period in China [61]. This had profound effects on the geographic distribution patterns of plants. However, our results showed that the area of suitable distribution for *A*. *chensiensis* was the largest during the LGM period and did not shrink as might be expected (Figure 4c, Table 2). On the contrary, the suitable distribution area expanded in a small range during the LIG period and shows a trend of retreat toward the southwest (Figure 4a). During the Quaternary period, species at high to mid-latitudes were affected by the spread of ice caps, while in low latitudes, due to drought and low temperatures [62], the geographic distribution of many species became fragmented, leaving many populations with an isolated distribution [63]. The relatively low latitude of China, with its complex topography and mountain barriers, prevented ice caps from covering southeastern China, which became one of the most important refuges for East Asian Tertiary relict gymnosperms to retreat southward during the Quaternary Ice Age [64,65]. Therefore, the distribution area of *A*. *chensiensis* was less affected by the glacial period during the LGM period and showed a tendency to migrate to the southeast and expand to the west.

### 4.3. Potential Distribution Area Changes and Conservation of A. chensiensis under Future Climate Scenarios

The simulation of future distributions showed that the suitable distribution areas of *A*. *chensiensis* will decrease under four different climate scenarios (RCP2.6, RCP4.5, RCP6.0 and RCP8.5) in the 2050s and 2070s. This phenomenon is also found in other rare and endangered plants, such as *Cathaya argyrophylla* and *Taiwania cryptomerioides* [66,67]. In the high emissions scenario for 2050, the temperatures rise by approximately 1.3 °C and are projected to rise to over 2.2 °C in 2070 [68,69], so the suitable range of *A*. *chensiensis* will generally decrease and habitat fragmentation will be more severe than at present. Under the RCP2.6—2070s emission scenario, *A*. *chensiensis* migrates 185,120 km to the southeast (Figure 6). Under the RCP6.0—2070s emission scenario, *A*. *chensiensis* migrates 24,772 km to the southwest (Figure 6). In addition, under the emission scenarios in the 2050s and 2070s, the migration routes of the centre of mass will migrate toward the southern high-altitude mountains (Figure 6a). However, the suitable distribution of species can involve biological, geological, or other disturbance factors in addition to the effects of climate change. Here, two plausible reasons may explain why the suitable distribution areas of *A*. *chensiensis* tend to the southern high-altitude mountains in the future. Firstly, it is mainly due to the growth habits of the species. The species is adapted to humid, warm climate and poorly tolerant of cold and drought characteristics (with an average annual temperature and annual precipitation of about 7.7 °C and 1347 mm in its range) [70]. As a result, it is difficult for the species to expand north of the Qinling Mountains due to temperature and precipitation constraints. Secondly, the species currently has a sporadic “island” distribution in the Qinba Mountains and their adjacent areas, as well as in southern Gansu (Figure 1). Recent studies have shown that the western Qinling Mountains are the Longzhong Basin and the northern Qinling Mountains are the Weihe Basin [71], and that these geographical barriers could impede large plant species from reaching suitable habitats [72,73]. So, we speculate that this may be one of the reasons why the suitable habitat for the species tends to be the southern high-altitude mountains in the future.

For a long time, *A*. *chensiensis* has been used as high-quality wood and medicine, and its natural population has been greatly harmed [25]. If not protected, coupled with ongoing climate change and the intensified impact of future human activities, the species is threatened with extinction, making conservation measures for *A*. *chensiensis* an urgent issue. The results of an ENMs study of *A*. *chensiensis* suggest that most current suitable areas will be suitable for this species under future climate change. Therefore, we recommend local protection of the wild natural population of this species, particularly by setting core protection areas at highly elevated regions of the Qinba Mountains and reducing the exploitation of this area. In addition, the mature seeds of each population can be collected and artificially sown to other populations by employing the cross-sowing method to improve the fragmentation of habitat, strengthen gene communication among populations, and improve the level of genetic diversity in wild populations.

## 5. Conclusions

In this study, the MaxEnt model was used to study and predict the distribution and shift of the potential suitable habitats of *A*. *chensiensis* in different periods. The ecological niche results show that the model could simulate the distribution range of *A*. *chensiensis* in China well. Environmental factors, such as Bio11, Bio5, Bio12 and Bio15, had a relatively great impact on the survival and distribution of *A*. *chensiensis*. From the past to the future, the core distribution area of *A*. *chensiensis* was relatively stable, mainly distributed in the Qinling–Daba Mountains, southern Gansu, and western Henan. Furthermore, in the 2050s and 2070s, the suitable distribution area will contract significantly; the centroids tend to migrate towards the southern high-altitude mountains. In response to climate warming, the core distribution area of *A*. *chensiensis* should ensure that it is not damaged and that it is protected in a targeted manner.

## Figures and Tables

**Table 1:** Description of environmental variables used in MaxEnt.

Type	Variable	Code Unit	Code Unit
	Temperature seasonality (standard deviation)	Bio4	°C
	Max temperature of warmest month	Bio5	°C
Climate	Mean temperature of coldest quarter	Bio11	°C
	Annual precipitation	Bio12	mm
	Precipitation seasonality (coefficient of variation)	Bio15	mm
	Precipitation of the coldest quarter	Bio19	mm

**Table 2:** Characteristics of potential distribution in different periods for *A*. *chensiensis*.

Period	Area of Each Suitable Region (×10^4^ km^2^)			
	Marginally Suitable Region	Moderately Suitable Region	Highly Suitable Region	Total Suitable Region
LIG	109.77	65.13	28.74	203.64
LGM (CCSM)	102.26	74.82	30.59	207.67
LGM (MIROC)	87.11	49.04	21.41	157.55
Current	96.04	48.48	35.38	179.91
2050RCP2.6	89.23	54.73	20.40	164.35
2050RCP4.5	109.55	56.82	19.06	185.43
2050RCP6.0	78.55	46.56	16.95	142.05
2050RCP8.5	94.42	45.59	23.03	163.04
2070RCP2.6	95.33	50.18	19.20	164.70
2070RCP4.5	89.30	49.51	9.63	144.44
2070RCP6.0	99.34	49.17	22.96	171.47
2070RCP8.5	95.14	46.82	31.20	173.16

**Table 3:** The potential distribution area of the *Abies chensiensis* species in the 2050s and 2070s.

Species	Period	Area of Each Suitable Region (×10^4^ Km^2^)			
		Unsuitable Region	Unchanged Region	Expansion Region	Contraction Region
*Abies chensiensis*	Current vs. RCP2.6-2050s	889.29	42.34	3.25	14.00
	Current vs. RCP4.5-2050s	894.40	37.14	8.45	8.90
	Current vs. RCP6.0-2050s	893.47	40.52	5.07	9.82
	Current vs. RCP8.5-2050s	897.51	36.61	3.81	8.86
	Current vs. RCP2.6-2070s	894.11	39.40	6.18	9.19
	Current vs. RCP4.5-2070s	887.94	41.85	3.74	15.36
	Current vs. RCP6.0-2070s	892.25	42.52	3.66	11.04
	Current vs. RCP8.5-2070s	891.08	39.23	7.24	9.24

## Data Availability

Not applicable.

## Supplementary Materials

The following supporting information can be downloaded at https://www.mdpi.com/article/10.3390/biology11111659/s1: Figure S1: AUC of current of *A*. *chensiensis* using ROC methods to test the results of MaxEnt; Figure S2: Results of Jackknife tests for the contribution of variables to the habitat distribution model of *A*. *chensiensis* for at present. The regularised training gain describes how well the MaxEnt distribution fits the presence data compared to a uniform distribution. The dark blue bars indicate the gain from using each variable in isolation, the light blue bars indicate the gain lost by removing the single variable from the full model, and the red bar indicates the gain using all of the variables; Table S1: species distribution record information; Table S2: percent contribution of each environmental variable.
