# Supplementary material for: Distributional Response of the Rare and Endangered Tree Species Abies chensiensis to Climate Change in East Asia
Source: Biology (Basel). 2022 Nov 13;11(11):1659. doi: 10.3390/biology11111659 (PMC9687575; doi:10.3390/biology11111659)
Supplement: Supplementary file 1 [file biology-11-01659-s001.zip › Table S2.pdf]

Table S2. Percent contribution of each environmental variable.

	**Period**	**Bio4**	**Bio5**	**Bio11**	**Bio12**	**Bio15**	**Bio19**
Percent contribution	Current	8.7	13.6	40.5	21.1	11.8	4.3
Permutation importance		3.3	6.9	58.5	15.9	11.6	3.7
