# Supplementary figures and images for: Distributional Response of the Rare and Endangered Tree Species Abies chensiensis to Climate Change in East Asia
Source: Biology (Basel). 2022 Nov 13;11(11):1659. doi: 10.3390/biology11111659 (PMC9687575; doi:10.3390/biology11111659)

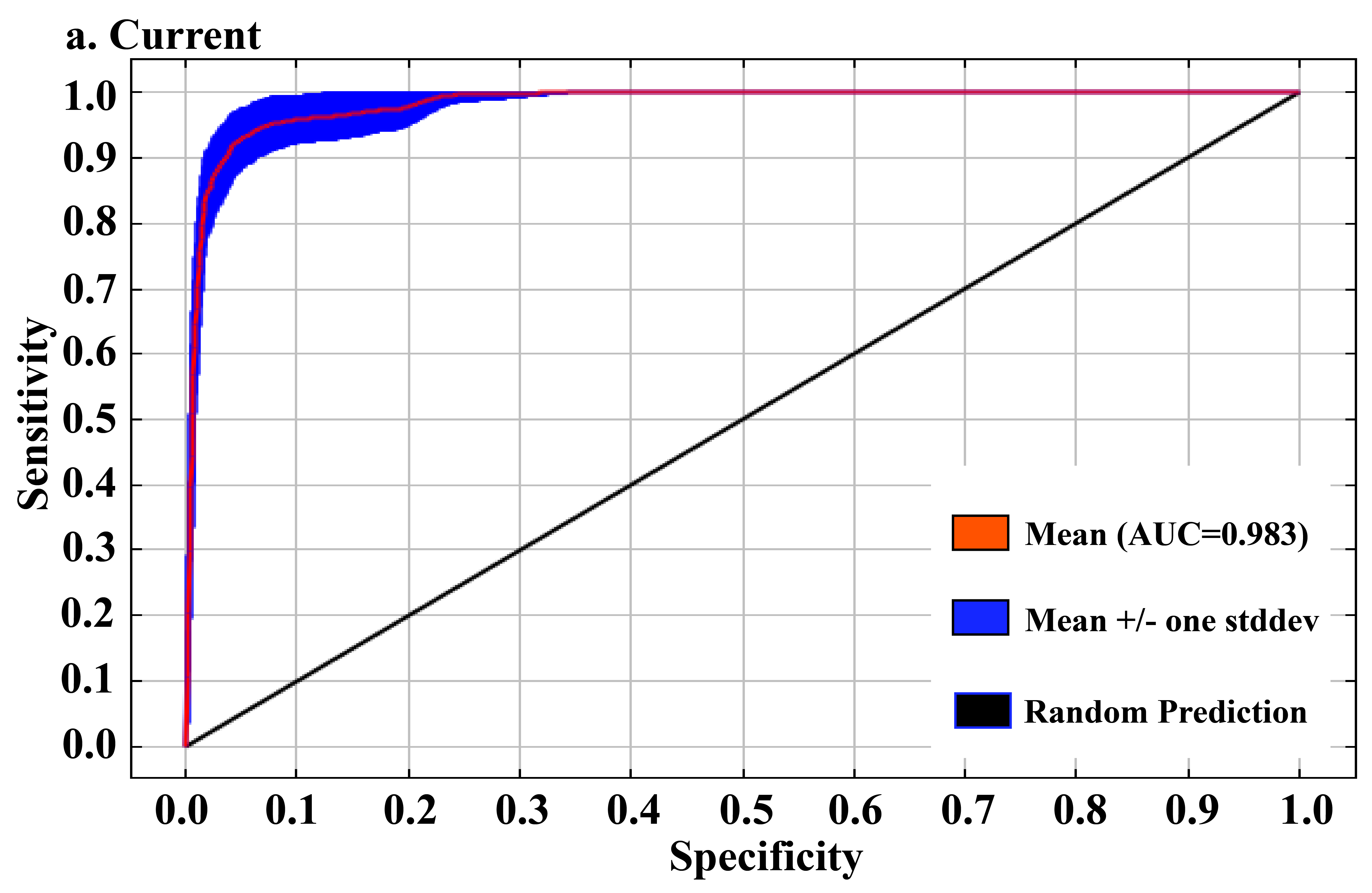

Supplement: Supplementary file 1 [file biology-11-01659-s001.zip › Figure S1.tif]

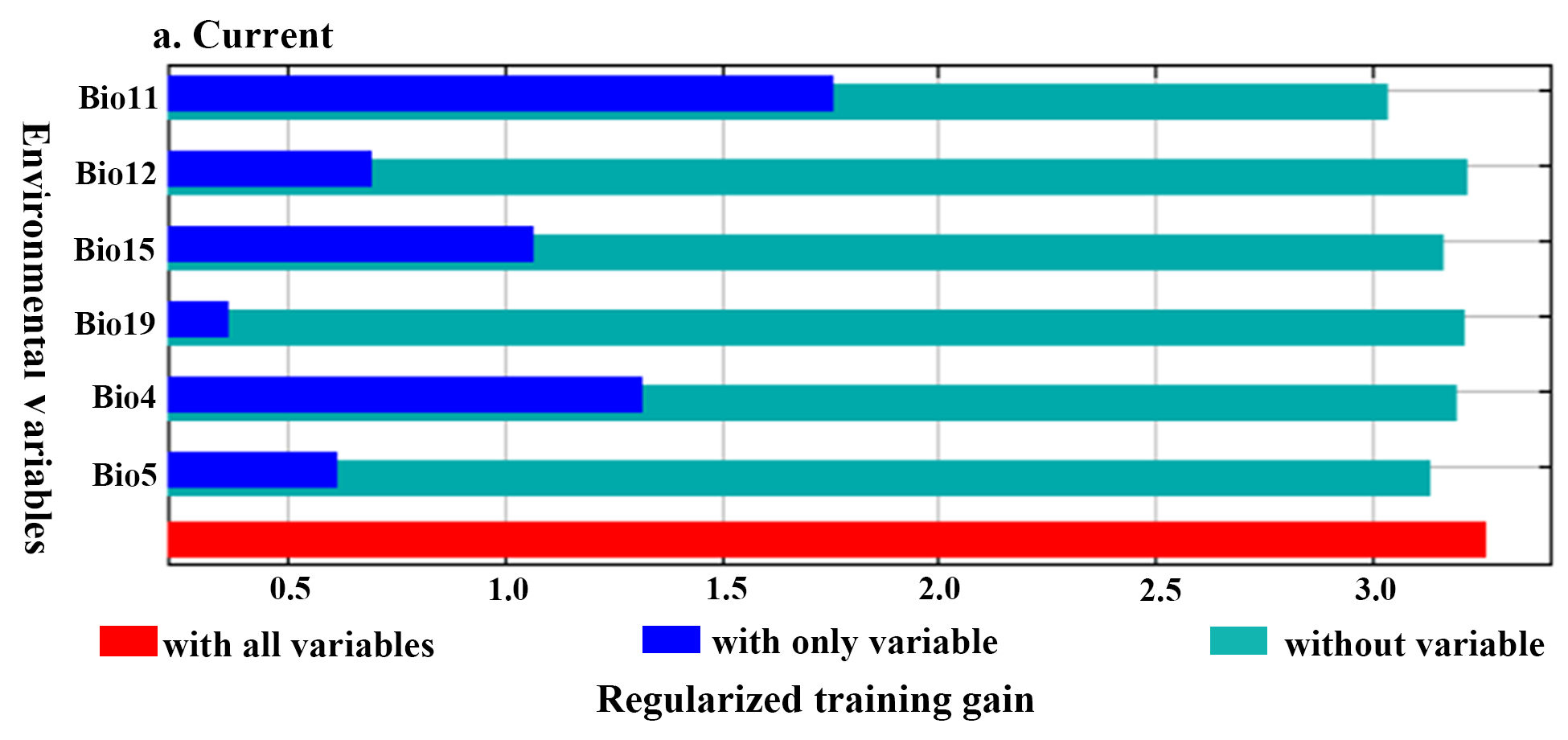

Supplement: Supplementary file 1 [file biology-11-01659-s001.zip › Figure S2.tif]
